# Human Endogenous Retroviruses and Diseases

**DOI:** 10.1002/mco2.70452

**Published:** 2025-11-02

**Authors:** Can Chen, Yanru Cui, Shixiang Wang, Yuze Yang, Zunpeng Liu, Suhan Jin, Fangqian Shen, Udo Gaipl, Hu Ma, Jian‐Guo Zhou

**Affiliations:** ^1^ Department of Oncology The Second Affiliated Hospital of Zunyi Medical University Zunyi Guizhou China; ^2^ Key Laboratory for Cancer Prevention and Treatment of Guizhou Province Zunyi Guizhou China; ^3^ Meinig School of Biomedical Engineering Cornell University Ithaca New York USA; ^4^ Department of Biomedical Informatics School of Life Sciences Central South University Changsha China; ^5^ School of Medicine Tsinghua University Beijing China; ^6^ Computer Science and Artificial Intelligence Lab Massachusetts Institute of Technology Cambridge Massachusetts USA; ^7^ Department of Orthodontics Affiliated Stomatological Hospital of Zunyi Medical University Zunyi China; ^8^ Department of Radiation Oncology Translational Radiobiology Universitätsklinikum Erlangen Friedrich‐Alexander‐Universität Erlangen‐Nürnberg Erlangen Germany

**Keywords:** human endogenous retroviruses; genetic domestication; transcriptional regulation; molecular mimicry; therapeutic targets

## Abstract

Human endogenous retroviruses (HERVs), remnants of ancient retroviral infections, comprise nearly 8% of the human genome and play dual roles in physiological regulation and disease pathogenesis. Once considered genomic “fossils,” HERVs are now known to dynamically influence gene expression, immunity, and homeostasis via epigenetic regulation, molecular mimicry, and viral mimicry. Their structural components, including long terminal repeats and conserved viral genes, enable them to act as regulatory elements and potential sources of novel antigens. However, the causal mechanisms linking the dysregulation of HERVs to diseases—the technical challenges in their detection and quantification, as well as their therapeutic potential—remain poorly systematized. This review synthesizes the molecular architecture and evolutionary trajectories of HERVs, emphasizing their tissue‐specific expression patterns. We further delineates their pathogenic roles in diseases including cancer, autoimmune conditions, and neurodegenerative disorders. Finally, we discuss emerging strategies targeting HERVs, including epigenetic modulators, immunotherapies, and gene editing, alongside ongoing clinical trials and translational challenges. By integrating molecular insights with clinical perspectives, this work provides a foundational framework for leveraging HERVs as biomarkers and therapeutic targets in precision medicine.

## Graphical abstract

Human endogenous retroviruses (HERVs), remnants of ancient retroviral integrations into the human genome, are modulated by viral infections, genetic inheritance, and triggers including environmental factors, epigenetic dysregulation, oncogene activation, and aging. Dysregulated HERV expression is implicated in cancers, neurodegenerative diseases, autoimmune diseases, and psychiatric disorders, highlighting its potential as a target for innovative therapies and clinical applications.

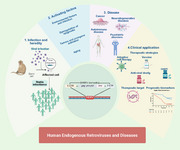

## 1 Introduction

Approximately 8% of the human genome comprises sequences derived from endogenous retroviruses (ERVs) [1, 2]. ERVs originate from ancient germline infections by exogenous retroviruses, which integrated their proviral DNA into host chromosomes [3]. Over time, these integrated sequences accumulated mutations and deletions, rendering them largely noninfectious but permanently embedding them within the genome [3, 4]. Human endogenous retroviruses (HERVs) are structurally characterized by flanking long terminal repeats (LTRs). These LTRs consist of identical nucleotide sequences, while the flanked internal region contains coding sequences homologous to retroviral *gag*, *pro*, *pol*, and *env* genes [5, 6]. Despite the loss of infectivity, ERV‐derived LTRs frequently function as regulatory elements, such as promoters and enhancers, capable of interacting with and modulating the transcription of nearby host genes [6, 7, 8].

In healthy tissues, HERVs expression is tightly constrained by epigenetic mechanisms, including DNA methylation at CpG‐rich promoter regions, histone modifications such as repressive H3K9 trimethylation and activating H3K27 acetylation, as well as chromatin remodeling complexes that modulate their genomic accessibility. These layered epigenetic controls collectively maintain HERVs in a predominantly silenced state under physiological conditions [9]. Analysis of global HERVs RNA expression profiles using multidimensional scaling revealed distinct expression patterns across tissues. Notably, brain subregions were most clearly differentiated, followed by tissues such as testis and adrenal gland, allowing them to be distinguished from other body sites. Furthermore, biological factors—sex, ethnicity, and age—significantly modulate HERVs expression across human tissues [10]. HERV transcription occurs through two primary mechanisms. First, it can occur via “leaky expression,” where RNA polymerase II reads through from actively transcribed host genes into adjacent HERVs elements, generating chimeric host‐HERVs transcripts. This passive process contributes significantly to baseline HERVs expression and exhibits cell‐type‐specific variability. Second, HERVs can be autonomously activated when their 5′ LTR promoters escape epigenetic silencing (e.g., via demethylation or viral trans‐activators), leading to stronger, locus‐specific transcription [10, 11, 12]. Although the emerging understanding of global HERVs transcription during homeostasis suggests the existence of many undiscovered roles for this activity, the extent by which HERVs RNAs may contribute to physiology remains unclear.

Genetics, environment, and age could affect cellular homeostasis [13, 14]. Chronic dysregulation of cellular homeostasis derepressed HERVs, resulting in pathologies characterized by inadvertent cytotoxicity [15], immunity activation, and/or cell senescence [16, 17, 18]. Deregulation of HERVs activity has been found in neurodegeneration, autoimmune inflammation, and oncogenesis [19, 20]. While strong correlative evidence from repeated observations underpins our current understanding linking HERVs activity to pathogenesis, establishing causal mechanisms through definitive mechanistic studies is now crucial. However, these studies are difficult to conduct owing to the discrepancies between the composition of ERVs in humans and in model organisms, and to the limited availability of reagents that accurately estimate the activity of HERVs elements.

This review aims to systematically collect the characteristics of HERVs at the molecular level, its pathological mechanisms in disease pathogenesis, and research advancements in the field of translational medicine. Through in‐depth analysis of the correlated and causal relationships between HERVs and complex diseases, we explore potential therapeutic opportunities embedded within these associations, thereby providing innovative insights for the diagnosis and treatment of related diseases.

## 2 Molecular Biology and Evolution of HERVs

HERVs are remnants of ancient retroviral infections that have become fixed in the human germline. Understanding their molecular architecture, evolutionary trajectory, and the mechanisms by which the host genome controls them is fundamental to deciphering their dual roles in physiology and pathology.

### 2.1 Structural Characteristics

**FIGURE 1:**
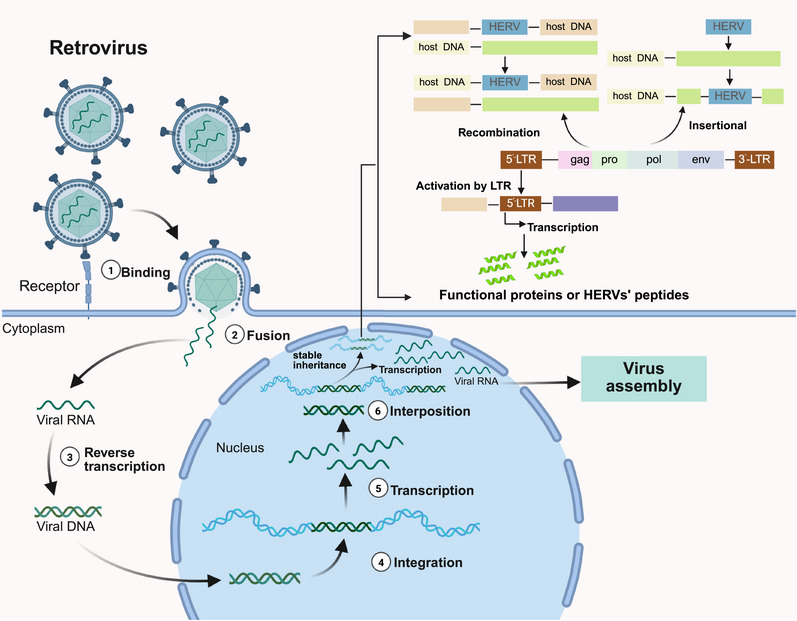
The retroviral infection cycle and provirus formation. The process of retroviral endogenization involves [25, 30]: ① and ② binding and fusion with the target cell, leading to the release of a capsid complex [248] containing RNA copies of the retroviral genome as well as integration factors (not shown). ③ Inside the cells cytoplasm, the viral RTases generate a DNA molecule from the retroviral RNA genome. ④ This DNA is subsequently transported into the nucleus for genomic integration, forming a provirus formation. Transcription and beyond (⑤ and ⑥): The integrated viral DNA (provirus) is recognized by the host cell's transcriptional machinery. This recognition triggers the transcription of the proviral DNA into viral RNA. These transcribed viral RNAs can have two main fates: they can act as the genetic material for the assembly of new retroviral particles, continuing the infectious cycle, or they may insert into and regulate host genes. This dual potential of viral RNAs highlights the complex interplay between retroviruses and their host genomes. Additionally, the diagram also shows the characteristics of HERVs (human endogenous retroviruses) in the upper right, including their presence in the human genome with multiple repetitive sequences, and processes like recombination and insertional events, as well as how they can be activated by LTR to produce functional proteins or HERVs’ peptides, further emphasizing the broader implications of retroviral‐related mechanisms in the context of host genetics. *Abbreviations*: HERV, human endogenous retroviruse; LTR, long terminal repeat. (Created with bioRender.com, with permission.)

HERVs have a typical proviral structure including LTRs sequences, located at both ends, containing promoters, enhancers, and polyadenylation signals, regulating the expression of viral genes [21]. Notably, some LTRs retain the capacity to drive aberrant transcription of adjacent host genes [22]. This structure encompasses two LTRs flanking four major genes (Figure 1): *gag* (containing matrix, capsid, and nucleocapsid domains), *pro* (containing protease and dUTPase domains), *pol* (containing reverse transcriptase, RNAse H, and integrase domains), and *env* (containing surface and transmembrane domains) [20, 23]. Beyond these canonical HERV structures, it is important to recognize that HERVs constitute a major class of LTR‐containing retroelements within the broader landscape of human endogenous retroelements [24]. This landscape also encompasses non‐LTR retrotransposons, which lack LTRs and utilize distinct retrotransposition mechanisms. Phylogenetic evidence, primarily from reverse transcriptase genes, suggests a shared ancestry between LTR‐containing and non‐LTR retroelements [24, 25], immunodeficiency virus type 1 (HIV‐1), and EBV [21, 26, 27, 28]. The LTR lengths, repetitive sequences and insertion sites of different families vary, and their recombination may cause genomic instability. The high heterogeneity of HERV structures is not only a “molecular fossil” of human evolution but also a source of potential pathological mechanisms epidemiologically linked to cancer, autoimmune diseases, and neurodegenerative diseases [26, 29]. Crucially, within the human genome, different retroelement groups—including various HERVs families—exhibit profound structural and functional heterogeneity. This encompasses variations in genomic architecture, replication autonomy, open reading frames integrity, and consequently, their capacity to generate retrotransposition intermediates or functional proteins [30]. Such inherent diversity underlies the differential capacities of specific retroelement groups to interact with host cellular pathways, notably the immune system [30].

Over extensive evolutionary time, the vast majority of HERVs have accumulated deleterious deletions or mutations, rendering them incapable of replication through the inactivation of these core genes [31]. However, certain evolutionarily younger HERVs subfamilies, particularly HERVK, HERVW, and HERVH, retain intact open reading frames for *gag*, *pol*, and* env*. This genomic integrity enables them to produce functional viral components and assemble virus‐like particles under specific permissive conditions. Reactivation occurs under permissive conditions including cellular stress (e.g., oxidative/hypoxic stress), epigenetic derepression via DNMT/HDAC inhibition, or during coinfection with exogenous viruses such as human.

### 2.2 Vertical Transmission and Expression Dynamics in Development

Vertical transmission is the defining mechanism by which HERVs became established as heritable genomic elements [32]. This process initiated when ancestral exogenous retroviruses infected primate germline cells, leading to reverse transcription of viral RNA into proviral DNA and its subsequent integration into host chromosomes via viral integrase (Figure 1) [33, 34]. Once fixed in the germline, these proviruses underwent Mendelian inheritance across generations [35]. Over evolutionary timescales, accumulated mutations inactivated most proviral ORFs, abolishing infectivity but permitting genomic domestication [29]. Crucially, preserved regulatory features—particularly LTRs with promoter/enhancer potential—and residual protein‐coding capacity enable HERVs to contribute functionally to host biology (Figure 1), underpinning their roles in development and disease pathogenesis.

The expression of ERV transcripts is detectable at the first cell division, indicating their early occurrence in embryogenesis. In humans, single‐cell transcriptomics reveals that distinct ERV families are dynamically activated in a lineage‐ and temporally resolved manner [36]. The HERVK subfamily, particularly the hominid‐specific LTR5Hs elements, peaks during the morula stage, coinciding with human embryonic genome activation [37]. Concurrently, LTR7/HERVH is highly expressed in the morula, while its subtype LTR7Y becomes enriched specifically in the epiblast lineage of blastocysts, marking pluripotency acquisition [37]. While HERVW elements are upregulated during trophoblast differentiation, aiding in the formation of the placenta [38]. This transcriptional burst is not stochastic but driven by the intrinsic regulatory capacity of ERV LTRs, which function as alternative promoters and enhancers for developmental genes.

During embryonic genome activation, evolutionarily young ERVs are activated through a conserved hierarchy: Krüppel‐like factors Krüppel‐like factor 4/Krüppel‐like factor 17 bind TEENhancers (LTR5Hs/HERVK, LTR7/HERVH, SVA), initiating chromatin opening via H3K27ac and forming naïve‐specific super‐enhancer hubs. These drive expression of proximal developmental regulators (e.g., ZFP42, ST6GAL1) through long‐range interactions—validated functionally by CRISPRi repression in naïve human embryonic stem cells. This activation is counterbalanced by rapid Krüppel‐associated box (KRAB) zinc‐finger proteins, which recruit KAP1 to deposit repressive H3K9me3 at cognate ERV loci. The resulting feedback loop enables precise taming of ERV‐derived elements, facilitating their exaptation into developmental networks while mitigating genotoxic risks [39].

ERV expression correlates with developmental plasticity. Murine 2C‐like cells—a rare metastable subpopulation in mESC cultures—reactivate MERV‐L, lose canonical pluripotency markers (OCT4, SOX2, NANOG), and regain the ability to contribute to extraembryonic tissues in chimeras [40, 41]. This state is stabilized by permissive chromatin (elevated H3K4me3, H3/H4 acetylation) and suppressed by epigenetic regulators (KDM1A, KAP1, G9a) that enforce pluripotency‐associated silencing [40]. In humans, the primate‐specific HERVH retrotransposon exhibits analogous associations with pluripotent states. Knockdown of HERVH RNA induces rapid differentiation, downregulates core pluripotency factors (OCT4, NANOG, SOX2), and upregulates lineage commitment markers [42]. The functional divergence between murine MERV‐L and human HERVH highlights species‐specific exaptation of ERVs. While MERV‐L reactivation enables murine totipotency reversion, HERVH sustains human pluripotency through chromatin looping and transcriptional scaffolding [40, 42]. Intriguingly, aberrant HERVH expression persists in pathological contexts such as glioblastoma stem cells, where related HERVK elements maintain stemness via OCT4‐mediated activation of LTR5Hs enhancers [43], suggesting conserved exploitation of ERV‐derived regulatory modules in developmental and disease plasticity.

Certain elements such as HERVH and HERVK may produce full‐length ERV transcripts, contributing to the transcriptome in distinct ways.Among the ERV family members, LTR elements of HERVH, HERVK, and HERVK (LTR 7, LTR 7Y, LTR 7B, LTR 5Hs, and LTR 14B) exhibit limited splicing based on the percentage of split‐RNA‐SEQ reads from ERV elements [37]. This suggests their involvement as promoters and transcriptional initiation or termination sites for HERVs. TEs also generate highly expressed lncRNAs in human pluripotent cells. Aberrant activation of HERVs has been implicated in triggering complex diseases such as multiple sclerosis (MS) and amyotrophic lateral sclerosis (ALS) [37, 44, 45]. However, there is mounting evidence indicating abundant expression of HERVs in normal tissues [46, 47, 48, 49].

**FIGURE 2:**
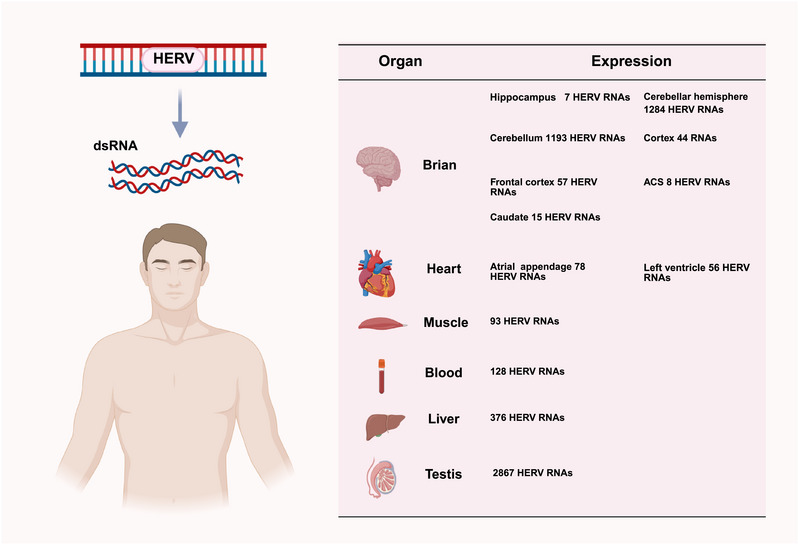
Tissue‐specific HERVs RNA expression profile. Detection of distinct HERVs RNA transcripts (numbers indicate identified RNAs) in major human organs, including brain, heart (atrial appendage and left ventricle), skeletal muscle, blood, liver, and testis [29]. *Abbreviations*: HERV, human endogenous retroviruse; dsRNA, double strand RNA. (Created with bioRender.com, with permission.)

**FIGURE 3:**
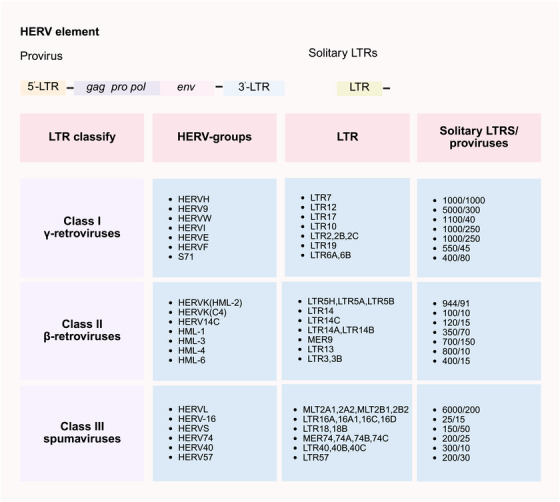
The diversity and genomic distribution of HERV components. The structure of HERVs provirus is depicted at the top, consisting of 5′‐LTR, gag, pro, pol, env genes, and 3′‐LTR. HERVs are categorized into three classes (Class I γ‐retroviruses, Class II β‐retroviruses, Class III spumaviruses) based on pol gene characteristics. For each class, associated HERV groups, representative LTRs, and the counts of solitary LTRs/proviruses (presented as [solitary LTRs/proviruses]) are listed. *Abbreviations*: HERV, human endogenous retroviruse; LTR, long terminal repeat. (Created with bioRender.com, with permission.)

Spatiotemporal patterns of HERVs activity during development are characterized by specific HERVs RNAs expression in anatomically distinct regions of the brain, testis, liver, skeletal muscle, blood, and heart (Figure 2) [10]. Notably, LTR12C significantly contributes to the unique HERVs expression profile observed specifically in the testis [10]. HERVs consist of proviruses containing specific genes surrounded by LTRs. Recombination events have resulted in isolated LTRs, but there are still around 4000 intact copies within human genome. Since the first HERVs was identified in the 1980s [50], over 3000 classifiable HERVs have been identified, and they can be divided into three classes (Class I, Class II, and Class III) and 11 supergroups based on the phylogeny of *pol* genes: Class I: MLLV, HERVERI, HERVFRDLIKE, HEPSI, HUERSP, HERVW9, HERVIPADP, MER50like, and HERVHF; Class II: HERVK/HML; and Class III: HSERVIII [51] (Figure 3). Each class is further divided into groups based on primer binding site sequences. Different groups within each class exhibit variations in sequence homology and distribution across the human genome.

### 2.3 Immune Recognition and Regulation of HERVs

**FIGURE 4:**
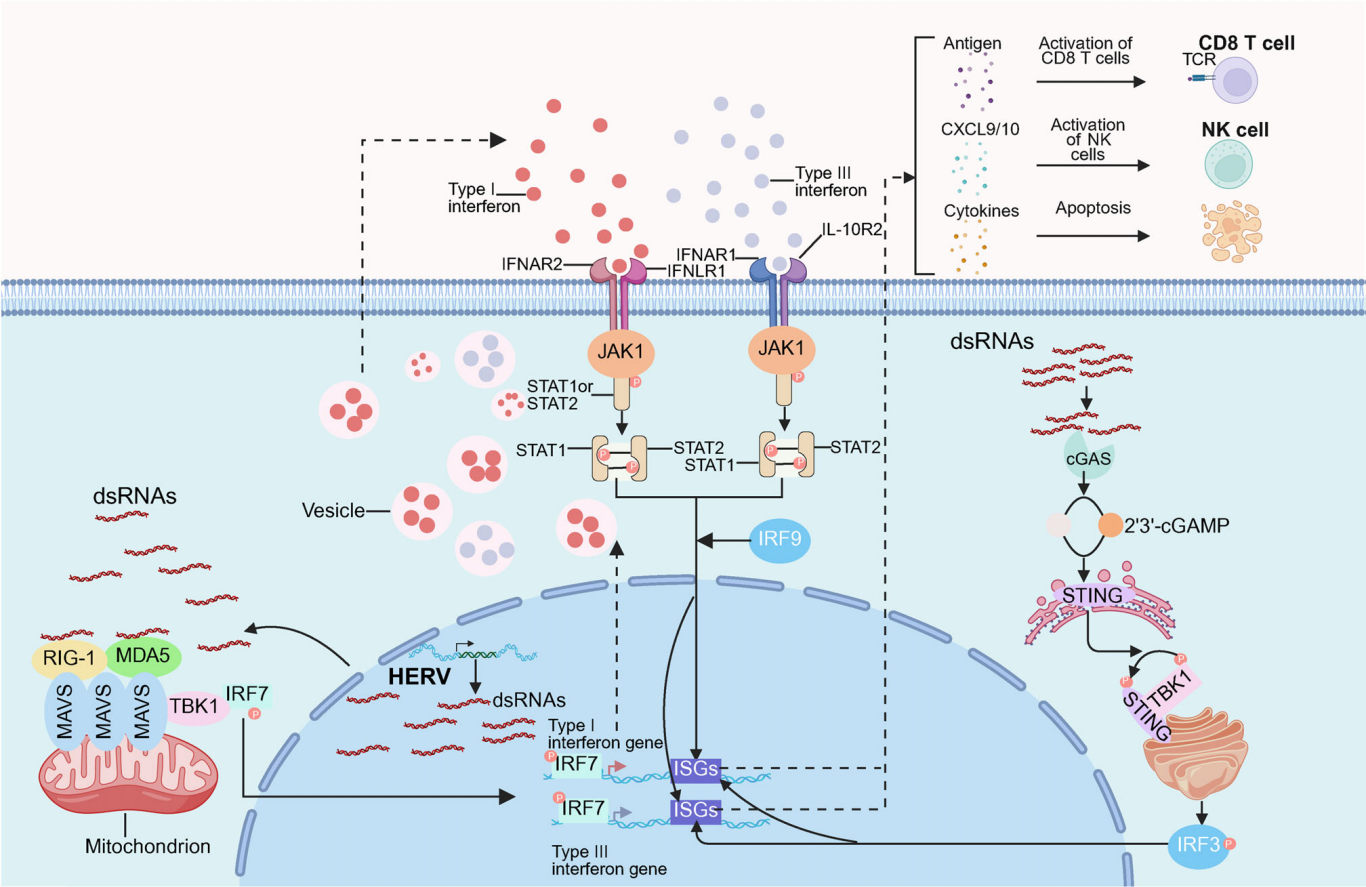
HERVs‐induced viral mimicry activates innate immunity. HERVs reactivation in cells may result in a viral mimicry state, causing in bidirectional transcription of ERVs to produce dsRNAs. These dsRNA are exported to the cytoplasm and recognized by PRRs, such as MDA5. MDA5 binding to dsRNA induces the recruitment of TBK1 and aggregation of mitochondrial antiviral signaling protein, which activate IRF7 through phosphorylation. Once activated, IRF7 translocates to the nucleus and induces transcription of IRG‐1. Consequently, type I/III interferons are produced, transported, and secreted into the tumor microenvironment. Secreted type I/III interferons enhance the expression of antigen processing and presentation mechanisms, thereby improving the ability of cancer cells to present antigens. Additionally, blebs from dying cell containing DNA and dsRNA are captured by DCs and also sensitizes cGAS in these cells. These pathways culminate in IFN‐I and IFN‐III production by both cancer and DCs, leading to efficient DCs activation and T cell priming, thus enhancing antitumor responses. *Abbreviations*: dsRNA, double strand RNA; PRRs, pattern recognition receptors; MDA5, melanoma differentiation‐associated protein 5; TBK1, TANK‐binding kinase 1; IRF7, interferon regulatory factor 7; IRG‐1, interferon‐responsive genes, DCs, dendritic cells; cGAS, cyclic GMP–AMP synthase; IFN‐I, type I interferon; IFN‐III, type III interferon. (Created with bioRender.com, with permission.)

HERVs engage in immune crosstalk through multiple pathways including human leukocyte antigen (HLA) class I‐mediated antigen presentation that activates cytotoxic T cells while suppressing NK cells via NKG2A [29]. Innate immune sensors detect HERV‐derived nucleic acids through distinct mechanisms: Toll‐like receptors (TLRs) discriminate ERV‐derived components as follows: TLR3 recognizes double‐stranded RNA (dsRNA), TLR7 senses single‐stranded RNA (ssRNA), and TLR9 responds to unmethylated CpG DNA; retinoic acid‐inducible gene I (RIG‐I) recognizes short dsRNA or 5′‐triphosphorylated RNA with duplex structures; melanoma differentiation‐associated protein 5 (MDA5) polymerizes on long dsRNA (e.g., >2 kb); and cytosolic DNA sensors (e.g., STING‐dependent pathways) sense reverse‐transcribed HERV DNA [52, 53]. This recognition triggers IFN‐α/β production via MAVS‐dependent (for RNA sensors) or STING‐dependent (for DNA sensors) pathways, activating downstream JAK–STAT signaling and upregulating interferon (IFN)‐stimulated genes to establish an antiviral state (Figure 4). Despite genomic integration, HERVs retain intrinsic retroviral features—including reverse transcriptase activity, (dsRNA) formation, and viral‐like particle assembly—that distinguish them from host genes.These features generate pathogen‐associated molecular patterns recognizable by the immune system [54]. Furthermore, specific HERV‐encoded proteins can directly modulate host cell functions, including upregulating intrinsic antiviral defenses and influencing RNA metabolism, thereby impacting processes like stem cell maintenance, oncogenesis, and neuroinflammation [29]. Collectively, the inherent “viral” characteristics of HERVs—including dsRNA formation, reverse transcription into complementary DNA (cDNA), and potential viral‐like particle assembly—render their replication intermediates and products intrinsically immunogenic, acting as key drivers of immune activation or dysregulation in various physiological and pathological contexts.

#### 2.3.1 Innate Immune Response to ERV‐Derived Nucleic Acids

The innate immune system employs specific pattern recognition receptors (PRRs), including TLRs (TLR3, TLR7, TLR9), RIG‐I‐like receptors (RIG‐I, MDA5), and the cytosolic DNA sensor cGAS, to detect nucleic acids derived from ERVs. This recognition triggers potent antiviral and proinflammatory responses [53, 55, 56]. TLR7 activation by endosomal ssRNA, notably ERV RNA in autoreactive B cells [55, 57, 58], critically contributes to the pathogenesis of autoimmune diseases such as systemic lupus erythematosus (SLE), with compelling evidence from both murine models and human patients [59]. Furthermore, genetic ablation of key components in the TLR7 signaling pathway suppresses the reactivation of endogenous retroviruses, exemplified by murine leukemia virus [60]. The cGAS–STING pathway senses cytoplasmic DNA, particularly cDNA derived from reverse‐transcribed ERVs, to initiate type I IFN‐I responses. This immunostimulatory axis has been demonstrated to drive T‐cell independent B‐cell activation and, in tumor contexts, mediates azacitidine‐induced ERV transcription‐dependent immune activation [61]. Notably, defects in nucleic acid metabolism cause pathological accumulation of endogenous nucleic acids, a hallmark of autoinflammatory syndromes such as Aicardi–Goutières syndrome (AGS) [62]. Furthermore, antiretroviral drugs alleviate inflammation by inhibiting the synthesis of ERV cDNA, but their nonspecific anti‐inflammatory effects suggest the complexity of the mechanism [63].

The expression of ERV, when simulating the viral environment, activates PPRs through its Env protein and dsRNA, forming a unique immune signal transduction network [24, 64, 65, 66]. IFN signaling establishes a positive feedback loop during this process: immune effector factors such as STAT1, IRF1, and NF‐κB can bind and activate the ERV promoter [24, 67, 68, 69, 70]. This leads to bidirectional transcription to generate dsRNA, and then activates type I/III IFN signals through TLR3 (endosome pathway) and MDA5–MAVS (cytoplasmic pathway), enhancing immunogenicity. It is particularly worth noting that IFNs can induce three types of “antisense” ERV transcripts and further amplify the innate immune response through the STAT1 signal [64, 67, 71]. This self‐reinforcing immune circuit is regulated by epigenetics under physiological conditions, but its abnormal activation may exceed the immune tolerance threshold and trigger an excessive immune response [72, 73].

#### 2.3.2 Adaptive Immune Response to ERV‐Derived Nucleic Acids

The complete ORF encoded by ERVs can produce antigens that can be recognized by T cell receptors (TCR) and B cell receptors, and participate in thymus selection to establish immune tolerance [74, 75]. The complete ORF encoded by ERVs generate endogenous self‐antigens, such as the HERV‐K18 superantigen and HERV‐W envelope protein [76, 77]. Constitutively expressed in human thymic double‐positive (DP) thymocytes, these antigens are presented by MHC class II molecules on thymic epithelial cells or hematopoietic antigen‐presenting cells [77]. This presentation drives negative selection of autoreactive thymocytes through TCR recognition, specifically deleting immature/semimature Vβ7+CD4+ T cell clones reactive to HERV‐K18 [77]. Consequently, peripheral Vβ7+CD4+ T cell levels inversely correlate with superantigen reactivity, establishing central tolerance to HERV‐derived epitopes. Incomplete deletion may permit low‐avidity clones to persist, contributing to autoimmunity when antigens are aberrantly expressed [74, 76, 78]. In addition, epigenetic silencing of ERVs in healthy tissues restricts their antigen presentation below the critical threshold needed to drive clonal deletion of reactive lymphocytes. Consequently, the immune system maintains a state of immune ignorance, characterized by the persistence of self‐reactive lymphocyte populations that remain functionally quiescent because of inadequate antigenic stimulation. This physiological state differs fundamentally from complete immune tolerance, where self‐reactive clones are either eliminated or actively suppressed [21, 29].

Under pathological conditions such as infection, inflammation, or tumorigenesis, disruption of this epigenetic repression (e.g., transactivation of human HERVs by the HIV‐1 Tat protein) can lead to re‐exposure of ERV antigens, thereby triggering adaptive immune responses. For example, spontaneous T/B cell responses against ERV antigens are associated with autoimmune diseases such as SLE [79], among which mouse models suggest that endogenous MLV antigens may drive the production of autoantibodies [56]. In cancer, widespread epigenetic dysregulation frequently results in aberrant expression of ERVs (e.g., HERVK). Their derivative antigens, which often belong to the cancer–testis antigen category, represent tumor‐specific antigens due to their silencing in most normal tissues [80]. Preclinical studies demonstrate that ERV antigen can activate antitumor T cells and mediate the rejection of transplanted tumors [81]. Strong T‐cell responses targeting the ERV epitope have also been found in human cancers, suggesting its potential for immunotherapy [82]. However, the immunogenicity of ERV antigens is double‐edged: they can be used to target tumors, but they may also exacerbate autoimmune pathology through molecular mimicry or cross‐reactions [83]. This mechanism provides a new direction for cancer immunotherapy, but its potential risks also need to be weighed.

### 2.4 Current Methods of Detection and Quantification

The detection and quantification of HERVs RNA face multiple technical challenges. Although its transcripts undergo processing procedures such as 5′ capping, splicing and 3′ polyadenylation, and can be captured by polyA enrichment for next‐generation sequencing the preparation of the library is susceptible to the quality of tissue preservation, RNA stability, and sequencing platform deviations [84]. The central challenge, however, stems from the high sequence homology among HERVs families. The extensive sequence homology results in a large fraction of sequencing reads mapping ambiguously to multiple genomic loci, confounding expression quantification. While long‐read sequencing technologies offer improved resolution by capturing unique structural motifs, their higher cost currently limits widespread adoption [85]. For the dominant short‐read sequencing approach, numerous bioinformatics tools have been developed to address the multimapping issue [86] (Table 1). At the single‐cell level, tools like scTE mitigate mapping errors by sacrificing precise locus specificity, whereas soloTE employs an expectation–maximization algorithm to retain locus‐resolved information [86]. In bulk RNA‐seq analysis, ERVmap enhances specificity by filtering reads overlapping known polymorphic sites within conserved regions [54]. Tools such as Telescope, SQuIRE, and SalmonTE reallocate fuzzy reads using probabilistic models, while HERVQuant relies on direct genome alignment to optimize quantitative accuracy [82]. However, there are significant differences in current HERVs annotations (such as the inconsistent classification criteria of databases like HERVd and Repeat Masker), and the calculation strategies of different tools vary, resulting in limited comparability of the results. There is an urgent need to establish a unified naming system, annotation standards and quantitative processes in the field to achieve the repeatability and cross‐platform integration of HERVs research.

**TABLE 1:** Comparison of bioinformatics tools for analyzing HERVs RNA sequencing data.

Tool	Alignment engine	Core algorithm	Input and workflow	Output and downstream analysis	MAPQ threshold	Optimal use case	Limitations	References
scTE (single‐cell)	STAR	Expectation‐Maximization (EM) with UMI deduplication	scRNA‐seq BAM + TE GTF → UMI collapse → probabilistic assignment	Single‐cell TE count matrix (raw counts), compatible with Seurat/Scanpy	≥255 (STAR default)	Single‐cell TE heterogeneity analysis	Lacks HERV subfamily resolution	[88]
soloTE (single‐cell)	STAR/HISAT2	k‐mer hashing with strand‐specific modeling	FASTQ → host+TE genome alignment → UMI clustering → TE quantification	Single‐cell TE count matrix (raw counts), compatible with Seurat/Scanpy	≥20	Strand‐specific scRNA‐seq (e.g., 10×5′ end)	Computationally intensive	[89]
ERVmap	STAR	Strict filtering (unique mapping + long fragments)	FASTQ → hg38_ERVmaster alignment → retain MAPQ ≥ 30 and length > 5 kb	High‐confidence ERV counts (DESeq2‐ready), with subfamily annotation	≥30	Clinical biomarker discovery	Potential loss of true signals and reference genome dependent	[54]
Telescope	HISAT2/STAR	Bayesian reassignment (Dirichlet prior)	BAM + TE BED → iterative read reallocation	Posterior probabilities per TE locus (TSV), supports differential expression	≥10	Dynamic TE activity profiling (e.g., tumor evolution)	Limited resolution in high‐homology regions	[90]
SQuIRE	Integrated STAR	EM algorithm with dynamic length normalization	FASTQ → automated alignment → coverage correction → locus‐level quantification	TE locus/subfamily counts and FPKM, RefSeq gene expression	≥20	Genome‐wide TE regulatory networks	Long runtime and custom annotation needed	[82]
SalmonTE	Pseudocounting	Lightweight k‐mer indexing (Salmon‐like)	FASTQ → direct pseudoalignment to TE reference → expectation–maximization	TE subfamily TPM values, compatible with DESeq2/edgeR	N/A	Large‐scale RNA‐seq screening	Dependent on high‐performance computing environments	[91]
hervQuant	Bowtie2/STAR	Hierarchical alignment (host‐first)	FASTQ → host alignment → unmapped reads → HERV library alignment → joint quantification	HERV family CPM matrix, limma‐compatible	N/A	Host‐HERV interactome studies	Suboptimal for chimeric reads	[92]

The table summarizes the technical approaches, advantages, limitations, and optimal use cases of seven computational pipelines designed to quantify HERVs expression from next‐generation sequencing datasets. Key challenges in HERVs RNA analysis include multimapping artifacts due to sequence homology, inconsistencies in HERVs annotations across databases (30–40% variability), and high computational costs (5–8× more expensive for long‐read sequencing). Future development should focus on hybrid machine‐learning/physical‐alignment methods, standardized HERVs databases (e.g., HERVGDB), and GPU‐accelerated implementations.

*Abbreviations*: EM, expectation‐maximization; TE, transposable element.

## 3 Regulation and Dysregulation of HERVs

HERVs are tightly controlled under homeostasis but can be reactivated through epigenetic dysregulation, viral infections, and aging‐related mechanisms. This section examines the multilayer control of HERVs, encompassing epigenetic silencing and transcriptional repression, and their pathologic derepression. Furthermore, it explores how the resulting nucleic acids and proteins contribute to pathogenesis via immune activation, genomic instability, and viral mimicry, thereby substantiating their functional roles in cancer, autoimmune disorders, and neurodegenerative diseases.

### 3.1 Mechanisms of Transcriptional Regulation and Dysregulation of HERVs

Recent research indicates that various environmental factors, including infectious agents, exogenous viruses, radiation, ageing‐related processes, epigenetic drugs, cytokines, and mitogens, can reactivate HERVs [50]. The regulation of HERVs expression involves intricate mechanisms encompassing multiple control strategies. Critically, these control systems, leading to aberrant HERV expression, is increasingly implicated in the pathogenesis of various diseases. Notably, emerging evidence demonstrates that HERV activity can be epigenetically modulated, for instance, via the PIWI‐interacting RNA pathway [21]. providing a mechanistic link between epigenetic silencing failure and pathological HERV re‐expression in disease contexts.

HERV‐derived promoters and genes can be actively transcribed and contribute to the regulation of gene expression across the genome [6, 93]. HERVs are regulated by multiple mechanisms, such as heterochromatin organization, DNA methylation, and histone methylation [1, 2, 94, 95, 96, 97, 98, 99, 100, 101, 102, 103, 104]. Disruption of the delicate balance between these endogenous retroviral elements and host cellular controls can trigger HERVs reactivation [105]. The activation of HERVs in cancer represents a complex process. Studies indicate that pathways involving DICER‐dependent small interfering RNA or antisense transcripts can suppress the expression of specific HERV families [9]. Epigenetic mechanisms, including DNA methylation and histone methylation, play crucial roles in regulating HERVs expression in both normal and cancerous tissues [9]. DNA methylation primarily silences evolutionarily younger HERVs with high CpG density within their LTR elements. Conversely, histone methylation is instrumental in silencing middle‐aged HERVs. Pharmacological agents targeting these pathways, such as inhibitors of DNA methyltransferases or histone methyltransferases, have proven effective in reversing HERVs silencing [106].

**FIGURE 5:**
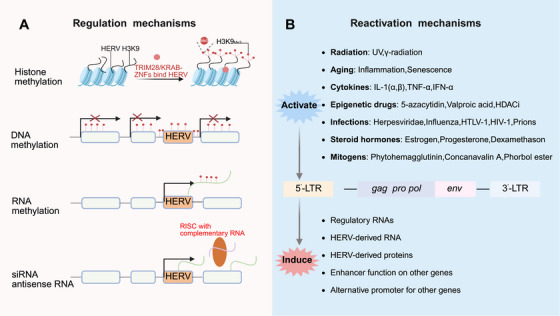
Regulation and reactivation mechanisms of HERVs. (A) Regulation mechanisms: HERVs can be suppressed at the level due, to mutations and are controlled by multiple layers of epigenetic regulation, resulting in limited transcriptional activity in most cell types. Key regulatory mechanisms include: (i) targeted heterochromatin formation, initiated by KRAB‐ZFPs binding specifically to HERVs sequences. This binding recruits TRIM28 (KAP1), serving as a scaffold to assemble heterochromatin‐associated proteins and epigenetic modifiers (e.g., histone methyltransferases, histone deacetylases); (ii) establishment of repressive epigenetic marks, primarily DNA methylation, which acts as a primary shield enforcing transcriptional silencing of HERVs loci in adult tissues; (iii) RNA‐mediated targeting also plays a role in inhibiting mammalian ERV. While some of these mechanisms are mainly observed in mice or different types of human transposon they may also play a role in controlling HERVs expression; and (iv) RNA‐mediated silencing pathways. While mechanisms like Dicer‐dependent RNA interference generating small interfering RNAs or antisense transcripts are well established for controlling specific TEs in mice and some human elements, analogous RNA‐directed silencing pathways likely contribute to HERVs regulation in humans. Collectively, these mechanisms tightly constrain HERVs expression. (B) Reactivation mechanism: The repressive state of HERVs can be disrupted by diverse exogenous and endogenous stimuli. Environmental factors (e.g., chemical agents, infectious agents), exogenous viral infections, radiation, aging‐associated processes, exposure to epigenetic‐modifying drugs, cytokines, and mitogens can alter epigenetic landscapes and/or signaling pathways, leading to HERVs reactivation. The revival of HERVs triggers the production of viral transcripts and proteins, which can impact host gene expression by acting as alternative promoters or enhancers. These occurrences could potentially influence crucial biological functions, in hosts or play a role in the onset of certain diseases. *Abbreviations*: HERV, human endogenous retroviruse; KRAB‐ZFPs, KRAB zinc finger proteins; TEs, transposable elements. (Created with bioRender.com, with permission.)

Furthermore, zinc finger proteins containing a KRAB domain establish repressive heterochromatin on HERVs. They achieve this by binding specific sequences within HERVs and recruiting the scaffolding protein TRIM28 (also known as KAP1). TRIM28 then facilitates the assembly of heterochromatin proteins and epigenetic modifiers, such as the histone methyltransferase SETDB1, to enforce silencing [21]. Specifically, the DNA methyltransferase inhibitor 5‐aza‐2′‐deoxycytidine (5‐aza‐CdR) has the potential to induce HERVs expression by reversing epigenetic silencing [106] (Figure 5A).

### 3.2 Extrinsic and Intrinsic Drivers of HERVs Derepression

HERVs’ repressive state is disrupted by exogenous/endogenous stimuli, causing transcriptional activation (derepression) via coordinated extrinsic/intrinsic drivers that destabilize host regulatory and epigenetic silencing systems.​ Extrinsic triggers include environmental/pathogenic stimuli: chemicals, pathogens (e.g., HIV‐1 Tat‐mediated transactivation) [27, 107, 108], radiation, inflammatory signals, epigenetic drugs, cytokines, mitogens, and oxidative stress—all perturbing host controls (Figure 5B) [21].​ Intrinsic mechanisms involve epigenetic reprogramming: dynamic histone modifications (e.g., NF‐κB‐dependent in inflammation) and DNA methylation changes [109, 110]. These, exacerbated by aging, synergize with extrinsic factors to break epigenetic barriers.​ HERV reactivation produces viral transcripts/proteins, which act as alternative promoters/enhancers to dysregulate host gene expression. This disrupts homeostasis, contributing to pathological processes in diseases like cancer and neuroinflammation. Understanding this interplay is key to defining HERV roles in health and disease.

#### 3.2.1 Viral Pathogens as Triggers of HERVs Dysregulation

The HIV‐1 Tat protein, as a key regulatory factor of the virus, not only activates the transcription of HIV itself, but also targets the LTR promoter region (e.g., U3 region) of HERVs by binding to host transcriptional extension complexes, thereby relieving epigenetic inhibition [27, 107]. Significantly enhance the transcriptional activities of families such as HERVK and HERVW. This cross‐activation interferes with the host immunity through multiple mechanisms: HERV Env proteins (e.g., Syncytin‐1) activate the TLR4 pathway and induce the release of proinflammatory factors (IL‐6, TNF‐α) [111]. The cross‐reaction between HERVs antigen and HIV epitopes may cause autoimmune damage. LTR reactivation can also lead to genomic instability through homologous recombination. Studies have shown that TAT‐mediated abnormal expression of HERVs is closely related to AIDS complications such as neurodegeneration and lymphoma, suggesting that targeting this pathway may provide a new direction for treatment [107]. Similarly, Epstein–Barr virus activates the NF‐κB pathway through the latent membrane protein LMP1/2A and upregulates the expression of HERVW/K [28]. The Tax protein of HTLV‐1 enhances HERVK transcription through the CREB/AP‐1 signaling, which may promote the malignant transformation of T cells [112]. Furthermore, influenza virus and HBV/HCV activate HERVK respectively through type IFN‐I and epigenetic dysregulation (e.g., DNA hypomethylation) induced by chronic inflammation [113]. Human hperpesvirus 6 inhibits the host silencing factor SETDB1 through viral miRNA to relieve the inhibition of HERVW [114]. The cytokine storm (e.g., IL‐6) caused by SARS‐CoV‐2 can upregulate the expression of HERVW/K and aggravate immunopathological damage [115]. These findings suggest that multiple viruses activate HERVs through direct (viral protein binding to LTR) or indirect mechanisms such as inflammation/epigenetic regulation, and their product, the Env protein, may jointly be involved in disease progression (e.g., neuroinflammation, cancer, and autoimmune responses). Therefore, targeting the HERVs regulatory pathway can provide a broad‐spectrum therapeutic strategy for complications of various viral infections.

#### 3.2.2 Endogenous Stressors as Activators of HERVs

Aging constitutes a potent endogenous stressor that progressively destabilizes epigenetic silencing mechanisms governing HERVs. During senescence, global erosion of heterochromatin—marked by diminished DNA methylation at CpG islands, reduced repressive histone modifications (e.g., H3K9me3), and impaired function of KRAB zinc‐finger proteins (KZFPs)—abolishes transcriptional suppression of retrotransposable elements, including HERVs [109, 110]. Crucially, the dioxygenase TET2 plays a pivotal role in mediating this age‐associated heterochromatin redistribution specifically in contexts of DNA methylation dysregulation, such as in aged hematopoietic stem and progenitor cells or upon DNMT loss‐of‐function, leading to ERV upregulation [116]. Concurrently, aging is associated with the induction of stress‐responsive transcription factors. Activating transcription factor 3 (ATF3), which is upregulated in senescence and aged tissues, directly binds to promoter/enhancer regions of a specific subset of ERVs termed senescence‐associated ERVs (SA‐ERVs). ATF3 binding, facilitated by the age‐related opening of chromatin (as evidenced by increased ATAC‐seq signal at SA‐ERV loci), acts as a key driver for their transcriptional derepression in senescent cells, aged human tissues, and progeroid models [117]. The reactivation of ERVs/SA‐ERVs generates dsRNA from HERVs transcripts, activating cytosolic sensors (e.g., cGAS–STING, MDA5, and TLR3) and triggering chronic IFN responses and neuroinflammation [109, 117, 118]. This chronic, low‐grade inflammation not only reflects the consequence of ERV reactivation but can also establish a deleterious positive feedback loop. Certain ERV subfamilies, such as ERVK, possess IFN‐stimulated response elements in their LTRs, making their transcription potentially responsive to IFN‐I signaling via IRFs, thereby further propagating their own expression and the inflammatory cascade [16]. This intricate interplay between age‐related epigenetic instability, stress‐induced transcription factors like ATF3, ERV reactivation (including specific SA‐ERVs), and innate immune hyperactivation represents a fundamental mechanism linking cellular senescence and organismal aging to the development of chronic inflammation and associated diseases.

## 4 Dysregulation of HERVs in Diseases

**FIGURE 6:**
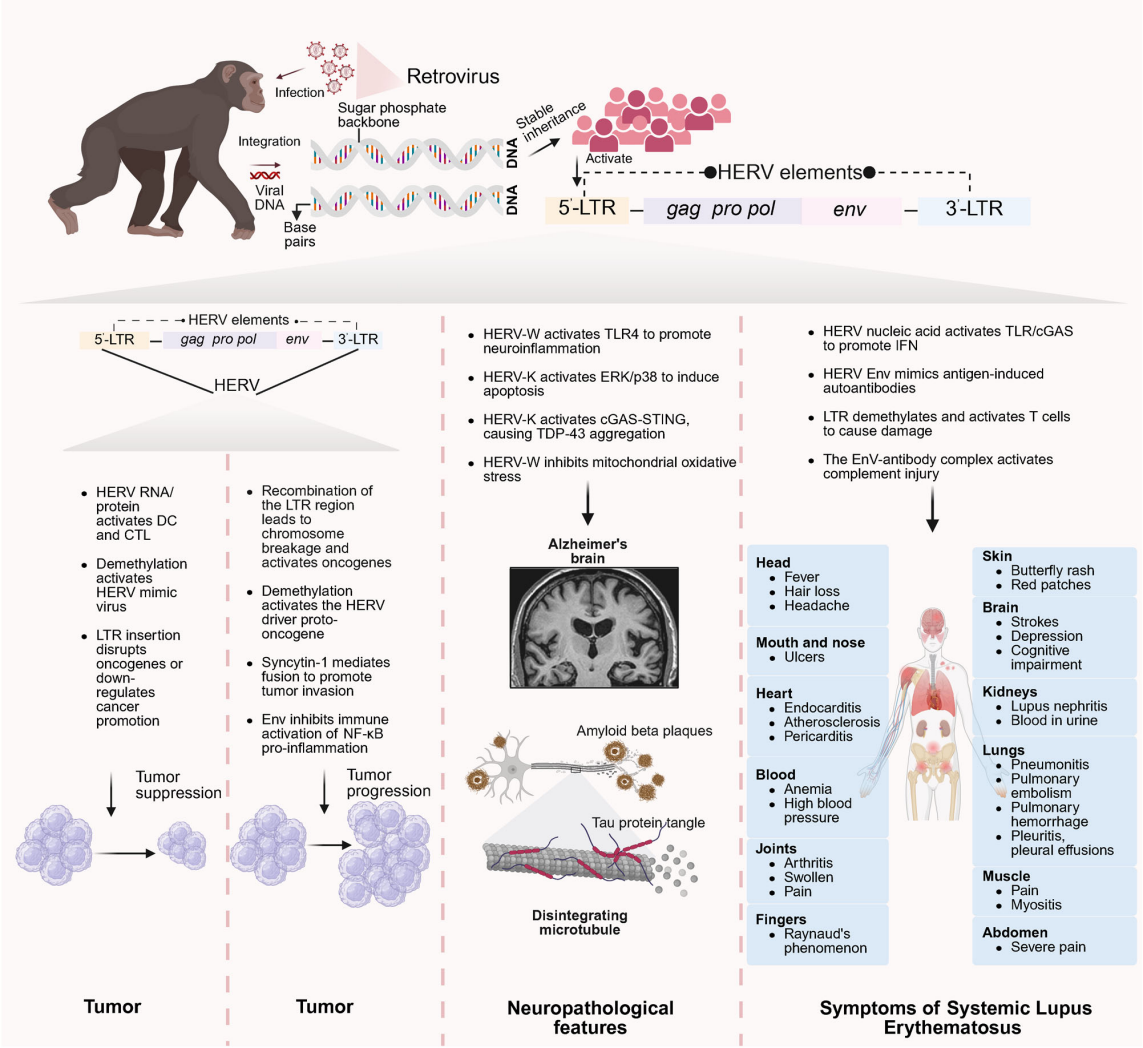
Pathological mechanisms of HERVs. This schematic summarizes the lifecycle of HERVs and their dysregulation in human disease. Ancestral retroviral infection of primates led to integration of viral DNA into the genome, with subsequent stable inheritance. HERVs elements, structured with 5′‐LTR, gag/pro/pol/env genes, and 3′‐LTR, can be reactivated in modern humans. Reactivation drives diverse pathogenic outcomes. Tumor: HERVs RNA/proteins modulate DCs and cytotoxic T‐lymphocyte function, while LTR insertions disrupt oncogene regulation, influencing tumor suppression or progression. Chromosomal recombination at LTRs and demethylation‐driven proto‐oncogene activation further contribute to tumorigenesis, alongside Env‐mediated NF‐κB inhibition and Syncytin‐1‐dependent tumor invasion. Neurodegeneration (e.g., AD): In AD, HERVW activates TLR4 to promote neuroinflammation; HERVK triggers ERK/p38‐mediated apoptosis and cGAS–STING‐driven TDP‐43 aggregation, while HERVW inhibits mitochondrial oxidative stress, intersecting with amyloid plaques and tau pathology. Autoimmunity (e.g., SLE): HERVs nucleic acids activate TLR/cGAS–IFN pathways; Env mimics autoantigens, and LTR demethylation activates T cells. Complement injury via Env–antibody complexes drives systemic manifestations spanning skin, joints, and organs. *Abbreviations*: HERV, human endogenous retroviruses; LTR, long terminal repeat; DCs, dendritic cells; AD, Alzheimer's disease; TLR4, Toll‐like receptor 4; ERK, extracellular regulated protein kinases; cGAS, cyclic GMP–AMP synthase; STING, stimulator of interferon genes; TDP‐43, TAR DNA binding protein‐43; SLE, systemic lupus erythematosus. (Created with bioRender.com, with permission.)

Accumulating evidence demonstrates that aberrant reactivation and expression of HERVs are implicated in the pathogenesis of diverse human diseases, spanning oncology, neurology, autoimmunity, and psychiatric disorders. This dysregulation manifests through complex mechanisms involving viral protein expression, genomic instability, epigenetic alterations, and immune modulation, contributing significantly to disease initiation and progression (Figure 6).

### 4.1 The Dual Role of HERVs in Cancer: Oncogenic Mechanisms and Immunotherapeutic Implications

#### 4.1.1 Oncogenic Mechanisms of HERVs

Tumorigenesis is a complex process that occurs through sequential mutations in cancer‐associated genes, including oncogenes, tumor suppressor genes, and genes involved in genomic instability [119, 120]. The aberrant expression of HERVs contributes to tumorigenesis through diverse molecular mechanisms, spanning protein‐mediated oncogenic signaling, genomic instability, epigenetic regulation, immune modulation.

HERVs have emerged as a pivotal driver of malignant progression, with HERVK exhibiting particularly potent dual oncogenic mechanisms. The Env of HERVK promotes tumor metastasis through ERK/NF‐κB signaling activation. This mechanism has been recently validated in ovarian cancer, where HERVK Env was found to increase chemosensitivity by inhibiting NF‐κB‐mediated upregulation of P‐glycoprotein [121]. Conversely, the *gag* protein compromises genomic stability by directly inhibiting p53 function—a relationship further elucidated by the identification of p53‐binding sites within HERVK's long terminal repeats (LTR5Hs), revealing a self‐reinforcing feedback loop wherein p53 impairment activates HERVK expression, thereby perpetuating oncogenic stress [122]. Importantly, HERVs RNAs form tumor‐specific antigens through abnormal splicing events (including exon‐transposition factor linkers), making them promising immunotherapy targets across multiple malignancies including bladder, kidney, and breast cancers [123, 124, 125, 126, 127, 128, 129]. Similarly pleiotropic, HERVW‐encoded Syncytin‐1 demonstrates context‐dependent oncogenicity, beyond its established fusogenic role in endometrial cancer, emerging evidence implicates Syncytin‐1 in immune evasion mechanisms, expanding its potential therapeutic relevance [130].

Growing evidence implicates HERVs as key contributors to cancer genome instability through two primary mechanisms, LTR‐mediated homologous recombination and retrotransposition‐dependent insertional mutagenesis. The highly conserved LTR sequences function as recombination hotspots, driving oncogenic chromosomal rearrangements [131]. Mechanistic studies reveal that epigenetically reactivated LTR12C elements induce double‐strand breaks and promote error‐prone repair via nonhomologous end joining [132]. Concurrently, active HERVK retrotransposition near oncogenic loci disrupts transcriptional regulatory networks. Single‐cell analyses further demonstrate HERV‐derived enhancer‐like activity in tumors and link HERVs insertions near cell cycle regulators to tumor metastasis and progression [133, 134, 135]. Together, these findings establish HERVs as multifunctional drivers of cancer genome evolution, operating through both structural and regulatory mechanisms.

Emerging evidence reveals how endogenous retroviruses orchestrate oncogenesis through interconnected RNA and DNA‐based mechanisms. The HERVH‐derived lncRNA network functions as a molecular sponge, sequestering tumor‐suppressive miRNAs to derepress MYC and KRAS oncogenes [136], a process amplified by hypoxia‐induced formation of phase‐separated biomolecular condensates that enhance miRNA binding capacity, as demonstrated by lncRNA–UCA1 in bladder cancer [137]. Complementing this posttranscriptional control, LTR elements remodel genomic architecture: (i) LTR7/HERVH mediates chromatin looping to activate CSF1R expression [138], while (ii) polymorphic LTR12C insertions create Krüppel‐like factor 4‐dependent super‐enhancers that aberrantly boost Wnt signaling in colorectal cancer. Notably, parallel work shows HERVK11 ncRNAs directly inhibit the tumor suppressor PSF [139, 140], releasing its brake on proto‐oncogenes—a mechanism shared with long‐spread nuclear elements‐1 elements that activate GAGE6 in liver cancer through PSF displacement [141].

HERVs exhibit a paradoxical duality in tumor immunity, functioning both as immunosuppressive mediators and sources of immunogenic neoantigens.The HERVK Env drives immune evasion by upregulating programmed death ligand‐1 (PD‐L1) on tumor and myeloid cells, inducing CD8+ T cell exhaustion—a process amplified by tumor‐derived exosomes that disseminate PD‐L1 as mobile “molecular mines” to systemically suppress T cell activity [125, 142]. Concurrently, IFN‐I and FNIII secreted by exhausted T cells initiates a self‐reinforcing cycle by reactivating dormant HERVK elements through JAK–STAT signaling and DNA demethylation, further elevating PD‐L1 expression [143]. Complementing this immunosuppressive axis, MHC‐I‐presented HERVs peptides promote tumor‐permissive inflammation through dysfunctional T cell responses and molecular mimicry [144]. Strikingly, these mechanisms converge in clear cell renal cell carcinoma, where hypoxia‐inducible factor HIF2α activates immunogenic ERV‐derived peptides capable of eliciting specific T cell responses, as evidenced by immunopeptidomics and allogeneic transplant studies [145]. This biological duality—coexisting immunosuppression and targetable immunogenicity—positions HERVs as both predictive biomarkers and therapeutic targets, with current clinical trials exploring Env‐blocking antibodies and personalized vaccines to exploit these mechanisms for cancer immunotherapy.

#### 4.1.2 HERVs as Biomarkers and Therapeutic Targets in Immunotherapy

HERVs expression signatures as potent predictors of therapeutic response across cancer types. In melanoma, high HERVK expression associates with superior immune checkpoint inhibitor response, attributable to viral mimicry‐induced T cell activation and IFN signaling [108, 146]. The biomarker potential exhibits tissue‐specific duality, estrogen receptor‐positive breast cancers with HERVW envelope expression show significantly worse progression‐free survival and intrinsic resistance to endocrine therapy through TLR4‐dependent NF‐κB pathway activation, whereas hypomethylated LTR12C elements in colorectal cancer patients' plasma achieve 92% specificity for early‐stage detection [147, 148]. These mechanistically informed biomarker associations are now entering clinical translation, with multiple ongoing trials evaluating HERV‐directed therapeutic strategies and their companion diagnostic applications.

T cell‐mediated immune surveillance forms the cornerstone of antitumor immunity, with immune checkpoint inhibitors against cytotoxic T lymphocyte antigen 4, programmed cell death‐1, and PD‐L1 demonstrating remarkable clinical efficacy across multiple malignancies [124, 149]. However, intrinsic and acquired resistance mechanisms limit their therapeutic potential [150, 151]. Enhanced expression of HERVs shows significant association with improved clinical response to ICI in ccRCC patients, positioning HERV‐derived signatures as promising predictive biomarkers for immunotherapy efficacy [142]. Consistent with this, integrated analyses of the CheckMate‐009, CheckMate‐010, and CheckMate‐025 clinical trials have pinpointed a novel nine‐ERV signature that serves as a specific prognostic indicator for overall survival in ccRCC patients receiving anti‐PD‐1 therapy [152]. Despite this considerable promise, key challenges persist. The immunogenic potential of HERV‐derived antigens requires further elucidation, particularly concerning their efficiency of antigen presentation and the immune evasion mechanisms they may encounter. This inherent biological duality underscores the critical need for precisely targeted therapeutic approaches capable of selectively harnessing the immunostimulatory properties of HERVs while mitigating their oncogenic contributions.

**FIGURE 7:**
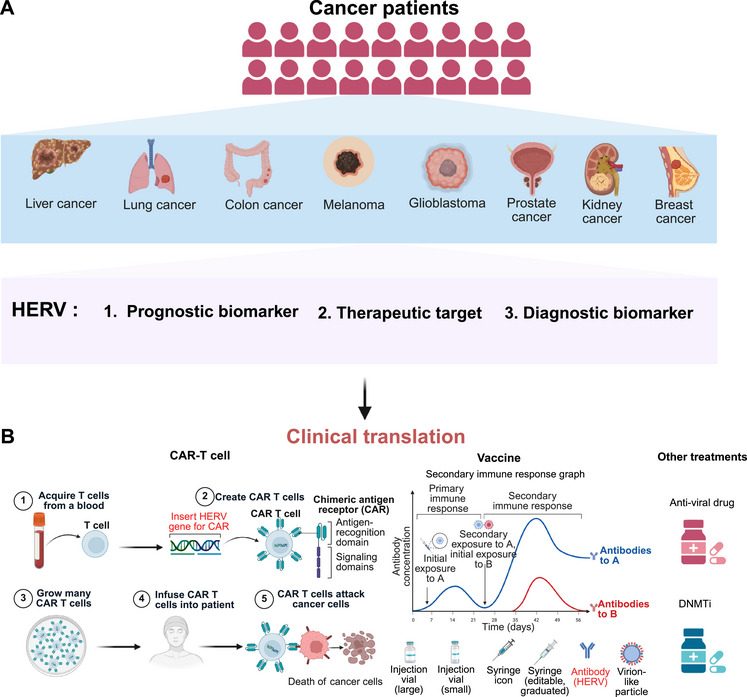
HERVs in cancer diagnosis and treatment. This schematic outlines the clinical utility of HERVs in cancer care. Panel A maps HERVs as prognostic, therapeutic, and diagnostic biomarkers across diverse malignancies (liver, lung, colon, melanoma, glioblastoma, prostate, kidney, breast cancers). Panel B details translation to interventions: CAR‐T therapy: T cells are engineered to express HERV—targeting chimeric antigen receptors, expanded, and reinfused to eradicate HERVs tumor cells. Vaccines: HERVs antigens trigger primary/secondary immune responses, leveraging memory B cells for sustained antitumor immunity. Other treatments: Antiviral agents and DNA methyltransferase inhibitors target HERVs activity to modulate tumor behavior. Collectively, it illustrates HERVs as a nexus for precision oncology, bridging biomarker discovery to therapeutic translation. *Abbreviations*: HERV, human endogenous retroviruses; CAR‐T, chimeric antigen receptor T cell; DNMTs, DNA methyltransferases. (Created with bioRender.com, with permission.)

HERVs, long‐regarded as genomic relics, are now emerging as pivotal players in cancer therapeutics. This visual framework underscores the translational potential of HERVs to revolutionize cancer treatment strategies. By leveraging HERVs as prognostic biomarkers, therapeutic targets, and diagnostic tools, novel interventions including chimeric antigen receptor T (CAR‐T) cell therapies, vaccines, and adjunctive treatments are being explored to combat a spectrum of malignancies. As highlighted herein, the integration of HERV‐based approaches into clinical oncology holds promise for advancing precision cancer care, with this schematic mapping the pathway from molecular insights to therapeutic applications (Figure 7).

### 4.2 HERVs Dysregulation in Autoimmune Diseases

Abnormal reactivation of HERVs represents a significant pathogenic mechanism driving inflammation in autoimmune diseases, acting as a crucial link between genetic susceptibility and environmental triggers [19, 79, 153]. HERV‐derived proinflammatory proteins and retrovirus‐like double‐stranded nucleic acids (dsDNA/dsRNA) can activate innate immune pathways, including cGAS–STING and RIG‐I, amplifying inflammatory cascades intracellularly [61]. This HERV‐mediated immune activation contributes critically to the pathogenesis of MS, SLE  [59, 154], psoriasis, and AGS [155, 156].

#### 4.2.1 Multiple Sclerosis

The link between retroviral elements and MS was first established in 1989 through analysis of leptomeningeal cell cultures from MS patients, initially termed MS‐associated retrovirus but later identified as HERVW [157, 158]. Subsequent research revealed that reactivation of these normally dormant sequences leads to expression of the HERV protein, which potently exacerbates immune responses [135, 159, 160, 161]. Elevated HERVW ENV RNA and protein levels have been consistently documented in the cerebrospinal fluid and serum of MS patients [162, 163, 164], with immunohistochemical studies demonstrating Env protein deposition in MS brains and its expression in myeloid, astroglial, and lymphoid cells [165, 166]. Importantly, herpesviruses—particularly EBV, recently implicated as a potential causative agent in MS—can activate dormant HERVW elements [167, 168, 169], suggesting a synergistic viral interplay in disease pathogenesis.

#### 4.2.2 Systemic Lupus Erythematosus

HERVs dysregulation contributes to SLE through three interconnected mechanisms: (1) nucleic acid sensing‐mediated immune activation, (2) molecular mimicry‐driven autoimmunity, and (3) epigenetic dysregulation of immune genes. Retroviral nucleic acids engage endosomal TLRs (TLR3/7/8/9) and cytoplasmic PPRs, amplifying IFN‐I responses characteristic of SLE [170, 171, 172, 173]. HERV‐encoded proteins can mimic self‐antigens, triggering cross‐reactive autoimmune responses. A key example involves the HRES‐1/p28 and HRES‐1/Rab4 proteins encoded by the HRES‐1/HERV. HRES‐1/p28 acts as an autoantigen, while HRES‐1/Rab4 disrupts CD3/TCRζ recycling in T cells, lowering their activation threshold [174]. Epigenetically, hypomethylation of HERVs LTRs derepresses adjacent immune genes, the MER41 LTR, for instance, serves as a STAT1‐binding promoter for AIM2, which shows decreased methylation and increased expression in SLE patients [67, 175]. Notably, HERVK ENV generate pathogenic immune complexes that induce nephropathy in models, while HERVE 4‐1 hypomethylation correlates with disease activity [176, 177]. These findings position HERVs as key mediators bridging environmental triggers with genetic susceptibility in SLE pathogenesis [174].

The pathogenic role of HERVs in SLE is further evidenced by clinical observations demonstrating elevated autoantibodies targeting HERVK102/K108 ENVs, whose titers show significant correlation with disease activity scores [178]. Parallel investigations identify HERVK‐derived small RNAs in peripheral blood mononuclear cells as promising diagnostic biomarkers, potentially enabling more precise disease monitoring [179]. From a genetic standpoint, comprehensive genomic analyses delineate a high‐risk profile combining low *C4A* gene copy number with increased HERVs genomic insertions—a configuration that confers substantial SLE predisposition regardless of clinical phenotype [180]. These findings collectively underscore HERVs elements as both immunological triggers and genetic modifiers in SLE pathogenesis.

### 4.3 Neurodegenerative Diseases

Emerging research has uncovered an unexpected player in neurodegenerative pathogenesis: endogenous retroviral elements. These genomically embedded sequences, long considered genomic “fossils,” are now recognized as potentially significant contributors to disease mechanisms across a spectrum of chronic, progressive central nervous system (CNS) disorders [181, 182, 183]. The clinical manifestations of these conditions—ranging from the motor neuron degeneration characteristic of ALS to the cognitive decline seen in Parkinson's disease (PD) and frontotemporal dementia (FTD), along with the cognitive impairment/dementia of Alzheimer's disease (AD) and certain psychiatric manifestations—belie remarkably complex and heterogeneous underlying etiologies. Intriguingly, the reactivation of these ancient viral elements appears to disrupt fundamental neural homeostasis through diverse mechanisms, potentially providing a unifying molecular framework that transcends traditional clinical classifications of neurological and psychiatric disease [184]. This paradigm shift suggests that endogenous retroviruses may represent both a novel diagnostic marker and therapeutic target across the neuropsychiatric spectrum.

#### 4.3.1 Amyotrophic Lateral Sclerosis

ALS represents a relentlessly progressive neurodegenerative condition characterized by the selective degeneration of motor systems, clinically manifesting as progressive impairment of gait, postural control, bulbar function (including speech and swallowing), and ultimately respiratory capacity [185]. Epidemiologically, ALS demonstrates a characteristic age of onset between 55 and 75 years, with a consistent global prevalence of four to eight cases per 100,000 individuals across populations [186, 187]. The uniformly poor prognosis, with median survival limited to approximately 3 years postdiagnosis primarily due to respiratory failure, highlights both the devastating nature of this disease and the critical need for novel therapeutic approaches targeting its underlying mechanisms.

Emerging evidence suggests a complex interplay between TDP‐43 pathology and endogenous retroviral activation in ALS pathogenesis. The hallmark cytoplasmic accumulation of TDP‐43 protein in motor cortex, corticospinal tracts, and anterior horn neurons [186, 188]. Appears mechanistically linked to dysregulation of HERVK activity. Biochemical studies demonstrate that TDP‐43 may function as a transcriptional activator of HERVs elements or disrupt retrotransposon silencing mechanisms, leading to aberrant HERVK expression [62, 189, 190]. This relationship exhibits striking bidirectionality—while TDP‐43 overexpression elevates HERVK env levels in neuronal models [15, 191, 192], targeted HERVK env silencing conversely modulates TDP‐43 expression [193], suggesting a pathogenic feedback loop. The downstream consequences of this interaction involve multiple neurotoxic pathways: TDP‐43‐mediated HERVs activation promotes DNA damage, caspase‐3‐dependent apoptosis [189, 194], and proinflammatory responses, with evidence of intercellular propagation of these deleterious effects. Clinically, elevated RT activity and HERVK env expression in ALS patient sera and CSF [192, 195] not only implicate retrotransposon activation in disease mechanisms but also highlight potential diagnostic applications. Importantly, while HERVK levels in neuronal extracellular vesicles show limited diagnostic value, their progressive elevation correlates with disease advancement [196], positioning them as potential prognostic biomarkers. These findings collectively position the TDP‐43/HERVK axis as a critical node in ALS pathophysiology, offering novel therapeutic targets for this devastating neurodegenerative disorder [197].

#### 4.3.2 Parkinson's Disease

Globally affecting ∼8.5 million individuals, PD ranks as the second most prevalent neurodegenerative disorder, with 1–2% prevalence among those aged over 65 years [198, 199]. As the fastest‐growing neurological condition, its prevalence is projected to double in the next two decades, posing significant societal challenges [200]. While most cases are sporadic (90–95%), 5–10% demonstrate familial inheritance patterns [201]. The disease is pathologically characterized by the selective loss of dopaminergic neurons in the ventrolateral tier of the substantia nigra pars compacta [202] and the presence of α‐synuclein aggregates (Lewy bodies) in both neurons and glial cells [201, 203]. These pathological changes underlie the hallmark motor symptoms (resting tremor, rigidity, bradykinesia) and debilitating nonmotor manifestations (cognitive impairment, autonomic dysfunction) [204]. Current therapies only address dopamine deficiency without modifying disease progression, underscoring the urgent need for mechanistic insights [203].

Emerging research implicates endogenous retroelements in PD pathogenesis. Postmortem studies reveal HERVs upregulation in prefrontal cortex and blood during advanced PD stages, with associated genes enriched in apoptosis and cellular respiration pathways [205]. While one study failed to detect HERVK pol transcripts in PD brains [206], genome‐wide analyses identified polymorphic HERVK elements potentially contributing to PD's neuroimmune phenotypes [207]. Notably, oxidative stress in substantia nigra pars compacta dopaminergic neurons correlates with long‐spread nuclear elements‐1 expression and retrotransposition events [208], and SINE–VNTR–Alu retrotransposons show association with disease progression [209]. Intriguingly, retroelement activation appears most prominent in symptom‐associated brain regions and could serve as a diagnostic biomarker. Recent evidence suggests viral particles may trigger PD‐like neuroinflammation, particularly in the presence of α‐synuclein fibrils [210], while HERVK env has been implicated in reactive astrogliosis [211]. These findings collectively suggest retroelements may contribute to PD pathophysiology through multiple mechanisms, though their precise roles require further elucidation.

#### 4.3.3 FTD and AD

FTD and AD similarly demonstrate retroelement involvement. FTD, characterized by behavioral and language disturbances [212], shows elevated serum and brain HERVK levels that colocalize with TDP‐43 inclusions [191]. In AD, the most prevalent dementia (50–75% of cases) [213, 214], tau pathology induces chromatin relaxation and retrotransposon activation [215, 216], with specific HERVs (particularly HERVK) showing marked upregulation.Transcriptomic analyses reveal associations between HERVs expression and neuroinflammatory pathways [217, 218], suggesting a potential vicious cycle where protein aggregates trigger retroelement dysregulation, which in turn exacerbates neuroinflammation and neurodegeneration [219, 220]. While these findings position retroelements as intriguing contributors across neurodegenerative diseases, their exact pathogenic mechanisms remain to be fully unraveled.

### 4.4 Psychiatric Disorders

Emerging evidence implicates HERVs in the pathogenesis of major psychiatric disorders, including schizophrenia, bipolar disorder, and autism spectrum disorder. A recent transcriptome‐genetic association study of 792 postmortem brain samples identified 26 cis‐regulated HERVs loci linked to mental disorders in European populations, with five high‐confidence risk loci specifically associated with schizophrenia, bipolar disorder, and depression. These HERVs display tissue‐specific expression patterns, supporting a potential causal role in neuropsychiatric disease mechanisms [221, 222, 223].

Notably, the HERVW Env was detected at significantly higher rates in patients with schizophrenia (41%) and bipolar disorder (28%) compared with healthy controls (4%). Patients positive for HERVW Env also exhibited elevated serum inflammatory markers, a history of childhood trauma, and more severe clinical symptoms, suggesting that HERVW may contribute to disease pathogenesis through epigenetic dysregulation and immune activation [224].

Mechanistic studies in transgenic mouse models demonstrate that HERVW Env expression induces cognitive deficits and disrupts synaptic function, including the downregulation of neurodevelopmental genes (Setd1a, Shank3). These effects coincide with aberrant histone methyltransferase activity (Kmt2a/b/d) and altered H3K4 methylation, implicating epigenetic misregulation in HERV‐mediated neuropathology. Strikingly, treatment with the LSD1 inhibitor ORY‐1001 reversed these epigenetic modifications and ameliorated behavioral abnormalities, providing direct evidence that HERVW Env drives neurodevelopmental dysfunction via epigenetic reprogramming [225].

These findings establish HERVW as a key mediator of neuropsychiatric disorders, acting through synaptic gene suppression, epigenetic remodeling, and neuroinflammation. Further research should explore whether targeted inhibition of HERVW or its downstream effects could offer novel therapeutic strategies for these debilitating conditions.

## 5 Therapeutic Strategies Targeting HERVs in Human Diseases

The recognition of HERVs as active contributors to pathogenesis, rather than inert genomic fossils, has catalyzed the development of novel therapeutic strategies. These approaches aim to silence aberrant HERVs expression, neutralize their pathogenic products, or exploit their unique properties for therapeutic benefit. Current strategies encompass immunotherapies (monoclonal antibodies, adoptive cell therapy), epigenetic modulators, reverse transcriptase inhibitors, and advanced gene editing techniques.

### 5.1 Clinical Trials of HERV‐Targeted Therapies: Outcomes and Hurdles

Once dismissed as genomic “fossils,” certain HERVs families (e.g., HERVW, HERVK) are now implicated in autoimmune disorders like MS and cancers through mechanisms ranging from molecular mimicry to epigenetic dysregulation. The table below synthesizes key interventional trials, highlighting targets, rationales, and outcomes that underscore the translational challenges and opportunities in this nascent field (Table 2).

**TABLE 2:** Key clinical trials evaluating HERV‐targeted therapeutic strategies.

Mechanisms	NCT number	Title	Status	Condition	Intervention	Characteristic	Population	Note
Reverse transcriptase inhibition pathway	NCT02437110	HERVK suppression using antiretroviral therapy in volunteers with ALS	Completed	ALS	Drug: darunavir; ritonavir; dolutegravir; TAF	Study type: Interventional Phase: phase 1 Primary purpose: treatment	Enrollment: 122 Age: 18 years and olders Sex: all	Unknown
	NCT05193994	Triumeq in amyotrophic lateral sclerosis (LIGHTHOUSE II)	Active, not recruiting	ALS	Drug: dolutegravir, abacavir and lamivudine; placebo	Study type: interventional Phase: phase 3 Primary purpose: treatment	Enrollment: 390 Age: 18 years and older (adult, older adult) Sex: all	No benefit observed in the interim analysis
	NCT01528865	Safety and efficacy of lamivudine and tenofovir to lower plasma level of viral RNA in lymphoma	Withdrawn	Lymphoma	Drug: lamivudine; tenofovir disoproxil fumarate	Study type: interventional Phase: phase 1 and 2 Primary purpose: treatment	Enrollment: 0 Age: 18 years and older (adult, older adult) Sex: all	Results are being updated
HERV envelope protein antagonism pathway	NCT02782858	Clinical trial assessing the HERVW Env antagonist GNbAC1 for efficacy in MS	Completed	MS	Drug: temelimab; placebo	Study type: interventional Phase: phase 2 Primary purpose: treatment	Enrollment: 270 Age: 18–55 years (adult) Sex: all	Positive results
	NCT03239860	Assessing the HERVW Env ANtagonist GNbAC1 for evaluation in an open label long‐term safety study in patients with MS	Terminated	MS	Drug: temelimab	Study type: interventional Study design: phase 2 Primary purpose: treatment	Enrollment: 220 Age: 18–55 years (adult) Sex: all	Results are being updated
	NCT05049161	A long‐term extension of study GNC‐401	Terminated	RMS	Drug: temelimab	Study type: interventional Phase: phase 2 Primary purpose: treatment	Enrollment: 33 Age: 18–55 years (adult) Sex: all	Unknown
	NCT04480307	Clinical trial assessing temelimab following rituximab treatment in patients with relapsing forms of MS	Completed	MS	Drug: temelimab	Study type: interventional Phase: phase 2 Primary purpose: treatment	Enrollment: 41 Age: 18–55 years (adult) Sex: all	Neutral results
	NCT03179423	Clinical trial assessing the GNbAC1 in patients with onset of type 1 diabetes within 4 years	Completed	MSRV	Drug: temelimab; placebo	Study type:interventional Phase: phase 2 Primary purpose: treatment	Enrollment: 64 Age: 18–55 years (adult) Sex: all	Positive results
	NCT05497089	Temelimab as a disease modifying therapy in patients with neuropsychiatric symptoms in post‐COVID 19 or PASC syndrome	Completed	Postacute sequelae of COVID‐19	Drug: temelimab; placebo	Study type:interventional Phase: phase 2 Primary purpose: treatment	Enrollment: 298 Age: 18–50 years (adult) Sex: all	Partial positive results
HERV‐specific immune targeting pathway	NCT03354390	HERVE TCR transduced autologous T cells in people with metastatic clear cell renal cell carcinoma	Active, not recruiting	Clear cell renal cell carcinoma	Cell infusion (HERVE TCR)	Study type: interventional Phase: phase 1 Primary purpose: treatment	Enrollment: 17 Age: 18–75 years (adult, older adult) Sex: all	Positive results
HERV epigenetic regulation observation	NCT02171884	Study of the impact of freezing‐thawing procedures and the prolonged culture of embryos on epigenetic regulation in humans	Completed	Assisted‐reproduction technology	Other: sample of cord blood; sample of placenta	Study type: observational Study design: observational Model: cohort time Perspective: prospective outcome	Enrollment: 298 Age: 18–50 years (adult) Sex: all	Results are being updated

The above summary of clinical trials is grounded in four distinct mechanisms of action [64, 81, 229, 230, 231, 232, 233, 234, 235], including antiretroviral therapies (e.g., darunavir/dolutegravir in ALS [NCT02437110] and dolutegravir/abacavir/lamivudine in LIGHTHOUSE‐II [NCT05193994]), monoclonal antibodies (e.g., temelimab for MS [NCT02782858] and COVID‐19 sequelae [NCT05497089]), and adoptive T‐cell therapy (HERVE TCR for renal carcinoma [NCT03354390]). Trials predominantly targeted neurological (ALS, MS) and immunological disorders (type 1 diabetes, PASC), with phases ranging from phase 1 to 3, enrollment sizes of 17–390 participants, and adult‐focused demographics (≥18 years). Notable outcomes include terminated studies (e.g., ANGEL‐MS [NCT03239860] due to safety/efficacy concerns, and a withdrawn lymphoma trial [NT01528865] with no enrollment). The heterogeneity in therapeutic strategies underscores both the potential of HERVs modulation and challenges in clinical translation—particularly the difficulty in achieving specific targeting of aberrantly expressed HERV elements without disrupting functionally critical endogenous retroviral sequences, the risk of off‐target immune activation due to cross‐reactivity between HERV antigens and host proteins, and the variability in HERV expression patterns across patient subgroups, which complicates the design of universally effective interventions—warranting further validation in controlled efficacy trials (clinical trials sources ‐Home | ClinicalTrials.gov).

*Abbreviarions*: ALS, amyotrophic lateral sclerosis; TAF, tenofovir alafenamide; MS, multiple sclerosis; RMS, relapsing forms of multiple sclerosis; MSRV, multiple sclerosis associated retrovirus; TCR, T cell receptor.

MS, the Env of HERVW has been implicated as a potential driver of neuroinflammation through aberrant immune activation. Mechanistic studies reveal that HERVW Env binds TLR4, triggering the activation of peripheral monocytes and central microglia. This interaction promotes the release of proinflammatory cytokines (e.g., IL‐6, TNF‐α) and enhances DCs‐mediated antigen presentation, thereby amplifying autoimmune responses against myelin [226]. Despite these findings, clinical translation has proven challenging. A phase II trial (NCT02782858) evaluating temelimab (GNbAC1), a monoclonal antibody targeting HERVW Env, demonstrated a reduction in serum HERVW RNA levels after 24 weeks of treatment in relapsing‐remitting MS patients [227]. However, no significant improvements were observed in key acute inflammatory markers, including MRI‐detected gadolinium‐enhancing T1 lesions or annualized relapse rates. This discrepancy suggests that HERVW Env may play a nonessential role in chronic disease stages, or that earlier intervention—prior to sustained neuroinflammation—is required for therapeutic efficacy.

Furthermore, emerging evidence indicates that HERVW may perpetuate neuroinflammation through Env‐independent mechanisms, such as dsRNA derived from reverse‐transcribed elements. These observations highlight the limitations of single‐target strategies and underscore the need for broader approaches to modulate HERV‐mediated immunopathology in MS.

In contrast to the limited success of HERVW Env‐targeted therapies in MS, RTase inhibitors have demonstrated more definitive therapeutic potential in AGS. This genetic disorder is characterized by cytoplasmic accumulation of dsDNA derived from endogenous retroelements, including HERVs, due to deficiencies in nucleases such as TREX1. The accumulating nucleic acids aberrantly activate the cGAS–STING pathway, leading to pathological IFN production (interferonopathy) [62], a 6‐month, open‐label phase II trial demonstrated that treatment with the RTase inhibitor lamivudine significantly CNS inflammatory markers (including cerebrospinal fluid IFN scores) in 60% of pediatric AGS patients, with concurrent improvements in motor function. Mechanistically, RTase inhibition is postulated to reduce the production of HERVs cDNA by blocking reverse transcription, thereby attenuating overactivation of innate DNA sensing pathways.

However, the therapeutic effects of RTase inhibitors must be interpreted with caution, as these agents simultaneously suppress other active retrotransposons, notably long interspersed nuclear element‐1 [228], this broad‐spectrum activity raises important questions regarding the relative contributions of HERVs versus other retroelements in AGS pathogenesis. While these findings underscore the value of targeting the reverse transcription process in IFN‐driven diseases, they also highlight the need for more precise characterization of disease‐specific retroelement profiles to optimize therapeutic strategies. Notably, the immunosuppressive microenvironment of solid tumors presents a major therapeutic challenge by limiting antigen accessibility and reducing the efficacy of conventional treatments. Emerging evidence suggests that targeting HERVs may offer a novel strategy to overcome these barriers. Notably, HERV‐derived tumor‐specific antigens are selectively expressed across multiple malignancies, including bladder urothelial carcinoma, clear cell renal cell carcinoma, colon adenocarcinoma, head and neck squamous cell carcinoma, lung adenocarcinoma, breast cancer, and lung squamous cell carcinoma [149].

Preclinical studies have demonstrated the therapeutic potential of HERV‐directed approaches. In lung adenocarcinoma and breast cancer models [127, 128], antibodies targeting the HERVK Env exhibit potent antitumor effects via humoral immune responses. These findings highlight HERVK Env as a promising target for immunotherapy. However, translating these observations into clinical applications requires addressing several challenges, including antigen heterogeneity and immune evasion mechanisms.The development of HERVK‐targeted therapies represents an ongoing exploration, with the potential to open new avenues for treating diverse cancers. Continued research is essential to optimize these strategies and harness the full immunogenic potential of HERV‐derived antigens in overcoming tumor immunosuppression.

### 5.2 Molecular Therapeutics for HERV‐Driven Pathologies: From Mechanisms to Drugs

#### 5.2.1 Gene Editing

Mounting evidence positions HERVK as a key regulator of glioblastoma stemness through its dual role in maintaining tumor stem cell niches and promoting therapy resistance. Mechanistically, HML‐2‐derived viral particles activate the TLR3/MyD88 pathway, subsequently driving IL‐6/STAT3 signaling to sustain stem cell self‐renewal and chemoresistance [43, 236]. This pathogenic axis can be effectively targeted through either CRISPR‐mediated silencing of LTR promoter activity [85] or pharmacological inhibition of reverse transcription (e.g., lamivudine), both of which demonstrate significant reduction in stemness markers (CD133, SOX2) and impairment of tumorigenic potential in preclinical models [55, 56]. These findings collectively establish HERVK as a therapeutically actionable target in GBM.

The development of CRISPR‐based interventions has yielded two distinct therapeutic paradigms: (i) transcriptional silencing approaches utilizing dCas9/KRAB or Cas9 systems to induce permanent epigenetic modifications or frameshift mutations in HERVK elements, with demonstrated efficacy in enhancing Programmed cell death‐1 checkpoint blockade in breast cancer models [80, 228], and (ii) synthetic lethality strategies that exploit HERVK‐associated vulnerabilities, including CRISPRa‐driven tumor‐specific expression of suicide genes (e.g., HSV‐TK) in melanoma or targeting of reverse transcriptase‐induced ATM repair defects in glioma [228]. These approaches highlight the versatility of CRISPR platforms in modulating HERVK activity across cancer types.

Key challenges moving forward include addressing HERVs subtype heterogeneity, mitigating potential off‐target effects on germline stability, and improving CNS delivery efficiency—particularly for brain malignancies. Future efforts should prioritize the integration of single‐cell epigenomic analyses to delineate tumor‐specific HERVs activation patterns [85], coupled with the development of precision editing tools that capitalize on LTR sequence polymorphisms. Overcoming these barriers will be essential for translating HERV‐targeted therapies into clinical applications for GBM and other HERV‐associated malignancies.

#### 5.2.2 DNMT Inhibitors

The virus–IFN–T‐cell activation axis, fundamental to antiviral immunity, is being strategically repurposed for cancer therapy through epigenetic manipulation. Hypomethylating agents that induce ERV reactivation create a state of viral mimicry, triggering robust antitumor immune responses [105, 237]. Notably, DNMT inhibitors demonstrate particular promise by upregulating HERVK expression, thereby stimulating immune signaling pathways that restore effector T‐cell function and overcome tumor immune evasion mechanisms. This approach forms a rational basis for combination therapies pairing epigenetic modulators with CAR‐T cells or HERVK‐targeted antibodies to enhance antigen‐specific tumor recognition [11, 238].

DNMT inhibitors exhibit concentration‐dependent pleiotropic effects, with low doses preferentially inducing gene reactivation while higher concentrations exert direct cytotoxicity [20, 239, 240]. Recent findings demonstrate that methyltransferase inhibition elevates both HERV‐derived RNA and DNA in cancer cells, generating endogenous nucleic acids that mimic viral infection.These molecular patterns are detected by cytoplasmic sensors, initiating innate immune responses that ultimately induce tumor cell apoptosis [241]. This epigenetically driven viral mimicry represents a particularly valuable strategy for targeting chemotherapy‐resistant malignancies.

The integration of HERV‐targeted epigenetic therapies with existing immunotherapies requires careful optimization of dosing schedules and biomarker development.Current challenges include managing the pleiotropic effects of DNMT inhibitors and identifying predictive signatures of response. Future studies should focus on delineating the specific HERVs elements that generate the most immunogenic responses, while exploring synergistic combinations with checkpoint inhibitors and cellular therapies to maximize therapeutic potential across cancer types.

#### 5.2.3 Antiviral Drugs

The repurposing of antiviral agents for cancer therapy represents an innovative frontier in oncology, driven by growing recognition of viral elements—particularly HERVs—in tumorigenesis and immune evasion. These agents, originally designed to combat exogenous viruses, now show promise in targeting viral‐like machinery encoded within cancer cells. HERV‐derived proteins and nucleic acids contribute to cellular transformation and metastatic progression, making them actionable targets. When combined with conventional therapies, antiviral strategies may address two fundamental challenges in cancer treatment: immunosuppression and therapeutic resistance, offering a multifaceted approach to tumor eradication [241].

While chemotherapy and radiotherapy primarily target proliferating cancer cells, their efficacy is often limited by tumor heterogeneity and acquired resistance. Antiviral agents introduce a complementary mechanism by disrupting viral components that support tumor survival. For example, reverse transcriptase inhibitors can suppress HERV‐mediated protumorigenic signaling, potentially sensitizing cells to cytotoxic therapies [242, 243]. This dual‐action strategy not only enhances direct tumor killing but may also mitigate resistance mechanisms, broadening the therapeutic window.

Beyond direct cytotoxicity, antiviral agents can remodel the tumor microenvironment by reversing immunosuppression. By inhibiting viral mimicry pathways or blocking HERV‐driven immune evasion, these compounds may restore immune surveillance and promote T‐cell‐mediated tumor clearance. When integrated with immunotherapies—such as checkpoint inhibitors or adoptive cell transfer—this approach could amplify antitumor responses, offering a rationale for combinatorial clinical trials [244]. Future research should focus on identifying predictive biomarkers to optimize patient selection and refine dosing strategies for maximal therapeutic benefit.

## 6 Disputes and Challenges

Substantial evidence links HERVs to human disease, yet their pathogenic roles remain debated. This section examines persistent challenges, including the limitations of current research, therapeutic targeting risks, and the evolutionary duality of HERVs. It also addresses technical impediments in HERV annotation and detection, highlighting the need for standardized genomic methods to enable mechanistic validation and clinical translation.

### 6.1 Disease Mechanisms: Beyond Correlative Evidence

The abnormal expression of HERVs are significantly associated with a variety of diseases. In malignant tumors, the HERVK subfamily is specifically and highly expressed in tumor tissues such as breast cancer and melanoma. By activating oncogenic signaling pathways such as MAPK/ERK and PI3K/AKT, it may directly promote the proliferation and metastasis of tumor cells. In the field of autoimmune diseases, elevated levels of the HERVW‐encoded capsule protein Syncytin‐1 can be detected in the cerebrospinal fluid of patients with MS. This protein can drive the neuroinflammatory cascade by activating TLR4 and inducing microglia to release proinflammatory factors such as IL‐6 and TNF‐α. In terms of neurodegenerative diseases, abnormal accumulation of HERVK transcripts has been found in the brain tissues of patients with AD. The dsRNA produced by it may accelerate neuronal degenerative changes by promoting the deposition of β‐amyloid protein and excessive phosphorylation of tau protein. However, most current studies are still limited to correlation analysis. The precise causal mechanisms through which HERVs contribute to disease pathogenesis—particularly regarding epigenetic regulatory specificity and the dynamic interplay between reverse‐transcribed elements and host genomic architecture—remain incompletely defined. It is necessary to further verify its pathological contribution by combining functional genomics and organoid models.

### 6.2 Focus of Scientific Disputes

The core of the controversy over HERVs activation focuses on three major aspects: the risk of viral revival, the side effects of therapeutic intervention, and the duality of evolutionary function. The long‐standing debate on the possibility of the “revival” of HERVs: It is believed that epigenetic dysregulation (e.g., DNA demethylation) may promote the formation of complete viral particles, but most HERVs are unable to replicate due to the loss of open reading frames. At the clinical application level, DNA methylation inhibitors widely used in cancer treatment (e.g., 5‐azacycloside) have been proven to activate HERVs. This “double‐edged sword” effect may enhance the therapeutic effect by strengthening the antitumor immune response, but the risk of simultaneously triggering an autoimmune response still needs to be vigilant. From an evolutionary perspective, some HERVs have been “domesticated” by the host as key physiological elements, such as the contribution of Syncytin protein to placental formation. This makes the targeted silencing of HERVs by gene editing technology face the dual challenges of functional disruption and ethical controversy. How to establish a precise regulatory mechanism between disease intervention and biosecurity, while balancing the “pathogenicity” and “functionality” of ancient viral remnants in the human genome.

## 7 Conclusion and Future Perspective

This review synthesizes HERVs as unique regulatory elements in the genome, play dual roles in development and diseases. During the embryonic period, they participate in cell fate determination through LTR epigenetic reprogramming (e.g., HERVK regulating trophoblast differentiation). In somatic cells, it affects the pathological process through chromatin conformational regulation (e.g., HERVH enhancing pluripotent genes) or immune interactions (e.g., HERVW activating the TLR4 pathway). However, its high heterogeneity (e.g., LTR sequence sharing rate >70%) and the limitations of detection techniques (only 10% of transcripts can be annotated) seriously hinder the analysis of causal mechanisms [17, 133, 236, 245, 246].

Clinical studies have revealed the potential mechanisms of HERVs in tumors, autoimmune diseases and neurodegenerative diseases. For example, in glioblastoma, HERVK maintains the characteristics of tumor stem cells by activating the TLR3/IL‐6/STAT3 axis through virus‐like particles. However, in the phase II trial of MS, although the HERVW‐targeted antibody (temelimab) did not improve acute inflammation, its chronic neuroprotective effect needs to be verified [247]. Epigenetic drugs such as azacitidine activate antiviral immunity by inhibiting HERVK transcription and show therapeutic potential in hematological malignancies and lupus erythematosus.

The core bottlenecks in clinical transformation include insufficient targeting specificity. Broad‐spectrum RTase inhibitors (e.g., lamivudine) simultaneously inhibit protective elements like long‐spread nuclear elements‐1, which may disrupt genomic stability. The risk of off‐target technology: when CRISPR edits HERVK, it mistakenly cuts the HERVW sequence, resulting in abnormal methylation of the host gene. Immunotherapy is inefficient, with low HERVs antigen presentation efficiency (e.g., the response rate of TCR‐T in the treatment of solid tumors is <30%) and is susceptible to the immune escape mechanism.

At the technical level, deep integration of gene editing (e.g., CRISPR/Cas9, base editing) with AI‐driven design is critical: machine learning algorithms can analyze sequence conservation and variation in HERVs families (e.g., 80–90% homology among HERVK subtypes) to precisely predict off‐target risks, design highly specific sgRNAs, and develop next‐gen high‐fidelity nucleases (e.g., Cas9 variants) to reduce nonspecific binding to homologous sequences. These tools should be paired with nano‐delivery systems (e.g., exosomes modified with RGD tumor‐homing peptides). By tuning particle size, surface charge, and targeting ligands, such systems can boost editing tool enrichment in solid tumor microenvironments, enhancing HERVK targeting efficiency by 3–5× in melanoma models while reducing accumulation in nontarget organs like the liver. Additionally, disease‐specific organoids (e.g., breast cancer, MS brain organoids) combined with single‐cell RNA sequencing and ATAC‐seq will dynamically track changes in genomic epigenetic modifications (e.g., LTR methylation), immune cell infiltration (e.g., T cell subsets), and signaling pathways (e.g., NF‐κB, PI3K/Akt) post‐HERVs intervention, enabling accurate preclinical validation of efficacy and risks.

In the realm of precision medicine, a cross‐disease HERVs expression profile database encompassing tumors, neurodegenerative diseases, and autoimmune disorders should be constructed, integrating patients’ clinical information (e.g., pathological stage, treatment response), HLA typing, and genomic data. Through multiomics association analysis, subtype‐specific biomarkers can be identified—for instance, high expression of HERVH in colorectal cancer serves as an independent indicator of poor prognosis, while the transcriptional level of HERVW in the cerebrospinal fluid of patients with MS correlates significantly with disease activity. Simultaneously, in‐depth investigation into the synergistic regulatory mechanisms between HERVs and immune checkpoints is warranted: HERVK‐derived antigens, for example, can enhance the immunogenicity of tumor cells by activating the STING pathway. When combined with PD‐1 inhibitors, the tumor regression rate in melanoma models can be more than doubled compared with monotherapy with inhibitors. Based on these findings, personalized therapeutic regimens can be designed, such as developing HERVK Env‐specific CAR‐T cells for patients with the HLA‐A*02:01 genotype, or formulating combination therapies comprising epigenetic modulators and immune checkpoint inhibitors tailored to leukemia patients with high HERVE expression.

At the ethical and translational interface, definitive boundaries for the clinical application of gene‐editing technologies must be delineated: germline editing is strictly prohibited, while a dynamic risk assessment framework for somatic cell editing should be established—incorporating long‐read genomic sequencing (e.g., PacBio technologies) to monitor HERV‐editing‐induced chromosomal structural variations (e.g., indels, translocations) and multiparametric flow cytometry to track immune tolerance profiles (e.g., autoantibody levels, Treg cell proportions). Standardization systems should be advanced through industry‐academia‐research consortia: unified protocols for HERV detection (e.g., benchmarked RNA‐seq data processing pipelines) must be formulated, and mechanisms for cross‐institutional sharing of biobanks and clinical databases established to accelerate the translational trajectory from basic research to preclinical validation.

Concurrently, international collaboration should be intensified: an open‐source HERV multiomics database (integrating genomic, transcriptomic, and proteomic data) and a transnational multicenter clinical trial network should be established to conduct adaptive clinical trials for indications such as HERVK‐positive melanoma and HERVW‐associated MS. The initial phase (phase I/II) will focus on safety validation, determining the optimal intervention window through dose‐escalation designs; the later phase (phase III) will verify the efficacy of combination therapies via randomized controlled trials. Ultimately, phased technological translation and multitherapy approval will be achieved, driving the paradigm shift of precision medicine from “pan‐targeted therapy” to “individualized virome regulation.”

## Author Contributions

Can Chen: writing—original draft, investigation, and formal analysis. Yanru Cui: writing—original draft, investigation, and formal analysis. Shixiang Wang: writing—original draft, investigation, and formal analysis. Yuze Yang: writing—original draft, investigation, and formal analysis. Jian‐Guo Zhou: writing—original draft, investigation, and formal analysis. Hu Ma: writing—original draft, investigation, and formal analysis. Udo Gaipl: writing—original draft, investigation, and formal analysis. Zunpeng Liu: writing—original draft, investigation, and formal analysis. Suhan Jin: writing—review and editing, supervision, formal analysis, and conceptualization. Fangqian Shen: writing—original draft, investigation, and formal analysis.All authors have read and approved the final manuscript

## Ethics Statement

The authors have nothing to report.

## Conflicts of Interest

The authors declare no conflicts of interest.

## Data Availability

No data were used for the research described in the article.
